# HOW IS MENTAL HEALTH IN LATE LIFE SHAPED BY STRUCTURAL RACISM?

**DOI:** 10.1093/geroni/igac059.1917

**Published:** 2022-12-20

**Authors:** Reed DeAngelis, Patricia Homan, Tyson Brown

**Affiliations:** University of North Carolina at Chapel Hill, Chapel Hill, North Carolina, United States; Florida State University, Tallahassee, Florida, United States; Duke University, Durham, North Carolina, United States

## Abstract

Less than one percent of studies on the link between race and health have focused on structural racism. Empirical research on how structural racism affects health in later life is especially rare. Moreover, the conceptualization of structural racism in the race theory literature has often differed from the measurement strategies used in aging and health research. This study advances the field by 1) utilizing a novel, theory-informed latent measure of structural racism in states across multiple domains, including political participation, education, economics, housing, and the judicial system, 2) mapping structural racism across states, and 3) quantifying the association between structural racism and mental health outcomes (depressive symptoms and frequency of poor mental health days) among Black and White older adults. We use administrative data measuring state-level racial stratification linked to geocoded individual-level demographic and health data from the HRS (N=9,126) and the BRFSS (N=308,029). Results show that, whereas structural racism is consistently associated with worse mental health for Black people, it is either unrelated to health or predictive of better health among Whites. Findings highlight the utility of rigorously conceptualizing and measuring structural racism and its impact on health among older adults.

